# Recovering phosphorus as struvite from anaerobic digestate of pig manure with ferrochrome slag as a magnesium source

**DOI:** 10.1016/j.heliyon.2023.e15506

**Published:** 2023-04-19

**Authors:** L.B. Moyo, G.S. Simate, T.A. Mamvura, G. Danha

**Affiliations:** a School of Chemical and Metallurgical Engineering, University of the Witwatersrand, Private Bag 3, Wits, 2050, Johannesburg, South Africa; b Department of Chemical Engineering, National University of Science and Technology, Box AC 939 Ascot, Bulawayo, Zimbabwe; c Department of Chemical, Materials and Metallurgical Engineering, Faculty of Engineering and Technology, Botswana International University of Science and Technology, Plot 10071, Boseja Ward, Private Bag 16 Palapye, Botswana

**Keywords:** Anaerobic digestion, Pig manure, Struvite, Ferrochrome slag, Response surface methodology

## Abstract

The circular economy initiative has driven the agriculture and agro-based industry to beneficiate from waste,thus closing the material loop towards enhancing economic and environmental performance. In this study, the aim was to recover phosphorus from anaerobic digestate of piggery wastewater (ADPW) using ferrochrome slag (FCSL) as the magnesium source to improve the environmental and economic sustainability of struvite precipitation. This was achieved by leaching 100 g L^−1^ of ferrochrome slag with 5 M HCl where 14.02 g L^−1^ of magnesium ions were extracted, and this acid-leachate of ferrochrome slag also contained 2650 mg L^−1^ of total iron. To simultaneously remove both high concentrations of organic matters in ADPW and iron in FCSL which are known to be detrimental to struvite precipitation, hydrogen peroxide (H_2_O_2_) at an H_2_O_2_/Fe molar ratio of 0.75 and pH 4.0 was added to the mixture of ADPW and FCSL. After the Fenton reaction, removal efficiencies of chemical oxygen demand (COD) and total iron reached 95.06% and 94.00%, respectively. Then COD and an iron-reduced mixture of ADPW and FCSL were added with a satisfying Mg:N:P molar ratio of 1.2:1:1.15 at pH 9.5 to produce struvite in 1 h. From 1 L of ADPW (2.6 g NH_3_–N), 0.5 L of FCSL (5.34 g Mg^2+^), and 6.62 g of PO_4_^3−^ P, were consumed to produce 45.57 g of struvite precipitate. Additionally, the economic feasibility of ferrochrome slag was assessed by estimating the chemical costs of FCSL against that of magnesium chloride which is commercially used. It was observed that using FCSL was cheaper as compared to using commercial MgCl_2_. Response surface methodology coupled with the central composite design was applied as a statistical tool to determine the effects of the key parameters (N:P; Mg:PO_4_; pH) on phosphorus recovery. Second-order polynomial equations were determined to correlate the parameters. ANOVA was applied and showed that p values for all the investigated parameters were less than 0.05 showing that they had a statistically significant effect on the phosphorus recovery. The study confirmed that it was possible to recover phosphorus as struvite from anaerobic digestate of pig manure with ferrochrome slag as a magnesium source.

## 1 Introduction

The growing concern for the longevity of global phosphate reserves has driven the need for exploring alternative phosphate sources [1]. Wastewater streams emanating from pig farms have been identified as one of the promising potential sources several nutrients including high phosphate content.In fact, waste beneficiation from these streams has been seen as an effective way of waste management since discarding these streams into nearby water bodies has proved to be problematic due to the negative effects such as eutrophication. Eutrophication has been shown to cause structural changes to the natural ecosystem such as increased production of algae and aquatic plants thereby depleting fish species and generally deteriorating water quality that reduce and preclude water use [2]. Consequently, sustainable management of these waste streams such as piggery wastewater through waste beneficiation is an important mitigation measure aimed at reducing the negative impacts of uncontrolled discharge of wastewater streams into water bodies.

One of the means of recovering phosphorus from pig slurry is through formation of struvite or magnesium ammonium phosphate (MgNH_4_PO_4_.6H_2_O) which is a slow-release mineral fertilizer. The formation of struvite requires at least an equimolar presence of Mg:N:P which is 1:1:1 [3]. Pig slurry generally has a high content of N and P but the levels of Mg are not sufficient for struvite precipitation to occur. This entails that an external source of magnesium is imperative for effective precipitation of phosphorus from the pig slurry as struvite. Conventionally, in commercial processes, mainly magnesium chloride (MgCl_2_), magnesium sulphate (MgSO_4_.H_2_0), and magnesium oxide (MgO) are much used but with distinct challenges [1]. Magnesium chloride (MgCl_2_) and magnesium sulphate (MgSO_4_.H_2_0) are expensive as compared to the other magnesium sources. However, magnesium oxide has operational problems as it is less soluble thereby requiring a longer contact time or an additional solubilisation step. On the other hand, seawater has been regarded as an effective alternative to the aforementioned conventional sources [4]; Aguardo et al., 2019). It has been envisaged that these low-cost magnesium sources such as seawater and bitten may reduce the production costs of struvite by 18–81% [3]. However, utilizing such sources for landlocked countries such as Zimbabwe will prove costly due to additional transportation and handling costs of the media from the point source.Therefore, this has driven the need to investigate readily available sources rich in magnesium that can be easily tapped and utilized in landlocked countries such as Zimbabwe.

Ferrochrome slag is such a resource in Zimbabwe, which is readily available and is a by-product from the production of ferrochrome an essential component in the stainless steel industry [5]. A substantial amount of ferrochrome slag is produced in Zimbabwe which is estimated to be between 1.1 and 1.6 tonnes of slag per tonne of ferrochrome produced [6]. Unfortunately, the associated generation of this solid waste in local mines is increasingly creating a burden on the environment as it occupies valuable landmass and is also creating a real challenge to its disposal as heavy metals are seeping into nearby water bodies. On the other hand, ferrochrome slag has found uses particularly in the construction industry as partial substitutes for cement or sand or added to clay and heat-treated to produce building materials such as ceramic tiles refractory and insulation blocks and as material in road construction [[7], [8], [9]]. These aforementioned uses have not been exhaustive. FCSL contains a high content of magnesium oxide (34-35 wt%). It is envisaged that this can be effectively used as a source of magnesium for struvite precipitation upon recovering the magnesium through acid leaching [10,11]. Silicon dioxide (SiO_2_) is an abundant component in ferrochrome slag which may not be a problem as it is converted to a gel-like silicic acid by a strong acid during the leaching process which can be easily removed by solid-liquid separation techniques.

Besides, iron oxide is one of the compounds found in ferrochrome slag, previous studies have shown that iron oxide is amenable to inorganic acids and the extent of acid leaching is in this order HF > HCl > H_2_SO_4_ [10]. As a result of using hydrochloric acid (HCl) for leaching the ferrochrome slag, it is anticipated that a substantial amount of iron (Fe) will be recovered in the leaching solution. Consequently, removal of Fe before precipitation of phosphorus is critical to obtaining a high-quality struvite product. According to Ref. [12] a ferric chloride coagulant can be produced from leaching iron ore tailings with HCl at moderate temperatures with a recovery rate of 94%. Similarly [13], successfully used a leached solution of ferronickel slag with comparable characteristics to ferrochrome slag as a coagulant in combination with oxidation with hydrogen peroxide of anaerobic digestate from pig waste. This process was effective in reducing colloidal particles as well as heavy metals before the precipitation process which compromised the quality of struvite. Physical separation techniques for treating colloidal wastewater such as sedimentation are not adequate since colloidal systems contain particles from 0.1 μm to 1 nm. This makes the settling velocity range from 0.3 to 3 m per year and implies that the processing time will be extremely long. Moreover, colloidal particles in wastewater tend not to agglomerate as they are stable. The main reason is that they possess an overall charge which makes the ions that surround them create a double layer so colloidal particles will repel each other preventing contact and agglomeration. In the Fenton reaction, ferrous and ferric ions react with hydrogen peroxide (H_2_O_2_) to produce hydroxyl (-OH) and hydroperoxyl (-OOH) radicals. These radicals strongly oxidize soluble and particulate organic substances and regenerated iron (Fe) species, inherently iron species, and colloidal particles are removed [14]. In addition, the presence of suspended material has been shown to result in an irregular morphology in the struvite crystals which compromises product quality (Wang et al., 2003). The critical parameters that will be investigated to optimize the coagulation process are the H_2_O_2_/Fe molar ratio and the pH. The H_2_O_2_/Fe molar ratio was chosen to be lower than in previous studies to avoid oxygen bubble formation due to the disintegration of residual H_2_O_2_ which will inherently destabilize settling particles.

On the other hand, a lower pH was avoided as this has been shown to produce carbon dioxide which is converted from dissolved inorganic carbon under strongly acidic conditions this will similarly have a negative effect as the aforementioned effects of the disintegration of residual H_2_O_2_. However, in a similar study by Ref. [13], a pH range between 2 and 4 was investigated and a pH of 4 was deduced to be optimal. This indicated that a higher pH range was required to be investigated. Previous research work has shown that at a lower concentration of iron chloride coagulant the removal of colloidal particles and soluble organic material is highly dependent on the pH with the optimum between 4 and 5.5 while at higher concentrations, the influence is less pronounced [15]. At a higher pH, there is a higher negative charge and reduced solubility of components in the system which hinders the coagulation process (Zhan et al., 2021). The optimal pH range is slightly lower than 5.8 where iron is the least soluble and exists as medium polymeric iron or monomeric iron which removes dissolved organic carbon by forming complexes and charge neutralization. Therefore, in this study, a pH range of 4–6 was investigated although at a pH of 6, Fe(III) has a limited solubility because of the precipitation of amorphous hydroxide which, on the other hand, can be critical for sweep flocculation aiding the coagulation process.

Despite this pre-treatment, the leached ferrochrome slag is envisaged to contain residual ions of iron as well as aluminium, chromium, and calcium which will interfere with the struvite precipitation. Generally, Ca ions compete with Mg ions for reaction with phosphate species. However, the interfering effect of these ions on struvite precipitation strongly depends on operational conditions. Therefore, this study not only aims to systematically evaluate the feasibility of using ferrochrome slag simultaneously as a coagulant but also to investigate the optimal conditions of using ferrochrome slag as a sustainable source of magnesium. Optimization of the process parameters in struvite crystallization is critical. The overdosing of components such as magnesium beyond the process needs increase the risk of uncontrolled struvite precipitation in other process stages and in the case of ferrochrome slag this results in unwanted residual ions in the system.

Response surface methodology (RSM) was adopted for the optimization of phosphate recovery from pig slurry. RSM enables the analysis of experimental data obtained from a set of experiments to optimize different variables which influence the desired response [16]. The experimental design was carried out using Central Composite Design (CCD). CCD is effective in evaluating the effect of all independent parameters and their interactions and identifies the optimum response in the least number of experimental runs. In this study, three variables pH, the molar ratio of N: P, and Mg: PO_4_^3−^ were chosen to study their effect on phosphate recovery.

## 2 Materials and methods

### 2.1 Ferrochrome slag leaching

The ferrochrome slag was collected from Zimasco, Kwekwe in Zimbabwe. It was initially pulverized and sieved to less than −75 μm. The mineralogy of the ferrochrome slag was determined using X-Ray Fluorescence (XRF) and is shown in Table 1.

**Table 1:** Mineralogy of ferrochrome slag (Dube et al., 2018).

Ferrochrome slag XRF analysis	
Compound	Average composition %
SiO_2_	31.3977
MgO	26.3939
Al_2_O_3_	18.0176
Cr_2_O_3_	14.3476
Fe_2_O_3_	4.4239
CaO	3.252
TiO_2_	0.556
Other	1.6111

The leaching experiments were performed in a 1 L vessel with an overhead variable speed mixer placed in a water bath to vary the temperature between 30 and 80 °C. A fixed amount of ferrochrome slag was used in all experiments maintaining a solid to liquid ratio of 1:10 with 500 mL of 5 M HCl used as the leaching solution. The contents were stirred at a constant rate of 250 rpm for 1 h. After leaching, the sample was filtered and thereafter analyzed for Mg concentration using atomic absorption spectroscope (AA-GBC Dual).

### 2.2 Pig slurry digestion

The raw swine slurry was collected from a commercial scale pig farm in (Bulawayo, Zimbabwe). Biological acidification of the pig slurry was achieved through co-digestion with banana peel waste in a 20 L anaerobic digester which was inoculated to achieve a high phosphate dissolution within four days to form the anaerobically digested pig waste (ADPW) [17]. Table 2 shows the characteristics of the raw pig manure.

**Table 2:** Feed Characteristics (g/L) (concentrations expressed per litre of raw pig manure).

TSS	VSS	Total-P	Total-N	Total-Ca	Total-Mg	pH	PO_4_–P	NH_4_–N	Ca^2+^	Mg^2+^
63.8	34.6	1.96	5.87	3.75	2.22	7.28	0.068	3.25	0.32	0.17

Table 3 shows the characteristics of anaerobic digestate pig waste before filtration.

**Table 3:** Characteristics (g/L) (concentrations expressed per litre of anaerobic pig waste digestate).

TSS	VSS	Total-P	Total-N	Total-Ca	Total-Mg	pH	PO_4_–P	NH_4_–N	Ca^2+^	Mg^2+^
60.7	29.8	1.571	2.805	2.488	1.198	5.4	1.571	2.805	2.488	1.198

### 2.3 Pre-treatment using Fenton reaction

The Fenton reaction was conducted in a batch reactor comprising a mixture of anaerobic digestate pig waste (ADPW) with characteristics shown in Table 3, and ferrochrome slag (FCSL), 1 L of anaerobic digestate pig waste was added into a 2 L glass beaker with a magnetic stirrer at 300 rpm. A solution of 28.0 wt% hydrogen peroxide was added into the mixture of anaerobic digestate pig waste and ferrochrome slag to adjust the molar ratio of H_2_O_2_ to Fe to 0.75 at a pH of 4 [13]. The concentration of hydrogen ions was adjusted using 5 M NaOH from a pH of 0. After the Fenton reaction, the mixture of anaerobic digestate pig waste and ferrochrome slag was filtered through a 0.45 μm Whatman filter paper, the residue on the filter paper was residual sludge. The filtrate was tested for chemical oxygen demand (COD), iron, magnesium, ammonia nitrogen and total suspended solids (TSS). The filtrate was thereafter used for struvite precipitation.

### 2.4 Struvite precipitation

Two litre containers were filled with the filtrate obtained after the Fenton reaction aforementioned. Thereafter, using 5 M NaOH the pH was adjusted to the desired alkaline conditions. The molar ratio of N:P was adjusted using potassium polyphosphate. Whereas, to satisfy the Mg:PO_4_ ratio the leached ferrochrome slag was utilized. An orbital shaker was used for mixing at 200 rpm for 1 h at room temperature. The formed precipitate was centrifuged at 3000 rpm for 10 min, washed with deionized water, and then dried at 40 °C for 24 h in a drying oven.

## 3 Results and discussion

### 3.1 Fenton reaction

**Fig. 1:**
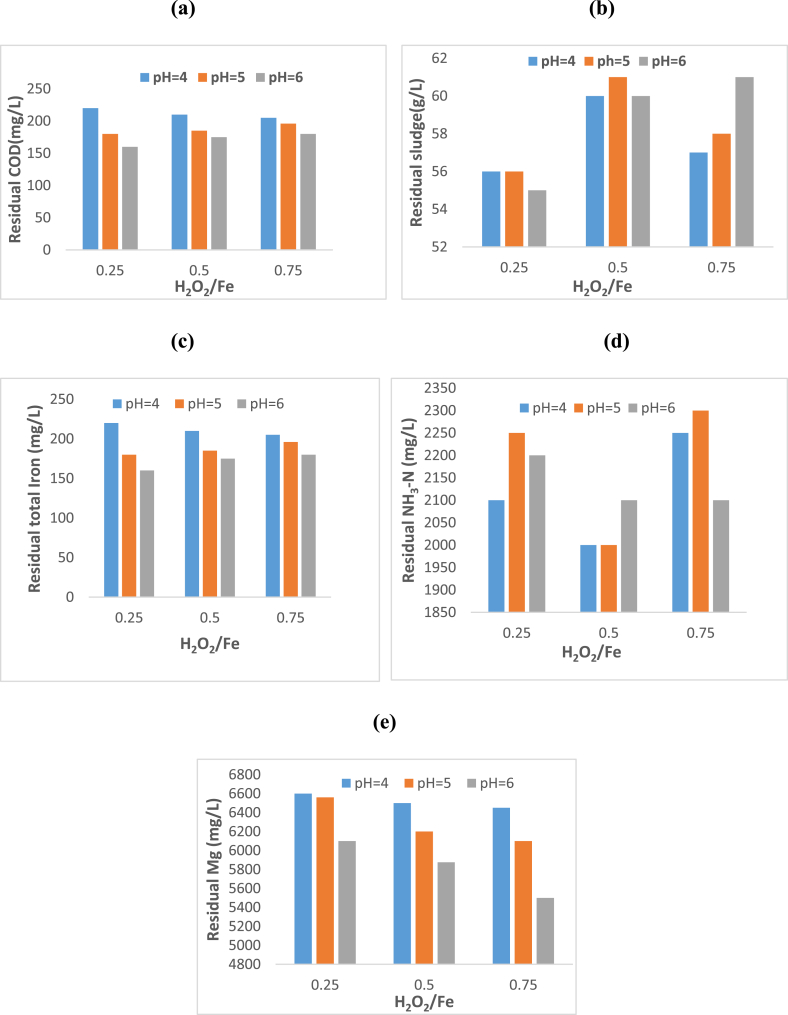
Effects of H_2_O_2_/Fe molar ratio and pH of Fenton reaction on residual concentration of (a) COD (b) residual sludge (c) iron (d) ammonia nitrogen (e) magnesium.

A pre-treatment process was imperative to reduce colloidal particles, iron (Fe), and dissolved organic matter in the mixture of anaerobic digestate pig waste and leached solution of ferrochrome slag, as these components are detrimental to the quality of struvite. H_2_O_2_ was used to induce the Fenton reaction. Fig. 1 shows the effects of Fenton reaction and coagulation at different pH and H_2_O_2_/Fe molar ratios on residual concentrations of COD, iron, ammonia nitrogen, and magnesium in the mixture of anaerobic digestate pig waste and ferrochrome slag. The initial concentrations of the aforementioned components are shown in Table 2. No bubbling was observed when the H_2_O_2_ was applied, this entailed that the concentration of the H_2_O_2_ used was not in excess but adequate for the oxidation process. Consequently, there was no residual H_2_O_2_ in the mixture to form oxygen bubbles upon decomposition of H_2_O_2_. A lower pH < 4 was avoided as carbon dioxide tends to form which is converted from dissolved inorganic carbon under acidic conditions [13]. The carbon dioxide will tend to interfere with the coagulation process as it will disperse settling particles.

Fig. 1a shows that at a lower H_2_O_2_/Fe ratio gave the highest residual COD concentration of 220 mg/L at a pH of 4 and this may be attributed to a small amount of organic material being oxidised by H_2_O_2_ as it is at low concentrations. On the other hand, the iron concentration at these conditions will be at its maximum possible concentration, this may mean an excess dosage of the coagulant which results in charge reversal and inducing a charge resulting in instability of particles. The mechanism involves iron particles hydrolysing rapidly but in a somewhat uncontrollable manner forming a series of metal hydrolysis species [15]. These species are multi-charged poly-nuclear complexes whereby in excess influence the charge density. In addition, Fig. 1a shows that COD was lowest at the highest pH. As pH increases the potential of iron to precipitate also increases. The precipitate formed facilitates sweep coagulation which results from the interaction of particulate matter with the iron precipitate as the particulate material is adsorbed onto the iron precipitate. Moreover, the flocs formed by charge neutralization which is predominant at lower pH levels are smaller than the flocs resulting due to sweep flocculation at higher pH. This combined effect of charge neutralization and sweep coagulation results in lower COD at a higher pH [18].

Fig. 1a and b also show that the amounts of COD and residual sludge across the H_2_O_2_/Fe and pH variations were comparable with a narrow range of 50–225 mg/L and 55–61 g/L respectively. This entails that the Fenton reaction and coagulation process were effective across the pH investigated. Fig. 1b shows that the highest residual sludge was observed at a H_2_O_2_/Fe molar ratio of 0.75 and pH of 6, which was coherent with the high reduction of COD observed in Fig. 1a. One of the drawbacks of using chemical coagulation is the large amount of residual sludge formed at the end of the process, in this study due to a combination of the Fenton and coagulation process, the amount of sludge formed in comparison to the initial amount of suspended material was not substantial.

Fig. 1c shows that the concentration of iron significantly reduced from the initial concentration of 2104 mg/L to 160 mg/L at pH 4 and H_2_O_2_/Fe ratio of 0.25, this shows that the coagulation process was not only effective in reducing suspended solids but also dissolved ions. Fig. 1d shows there was a small change in the ammonia-nitrogen concentration compared to the other components under investigation. Whereas, Fig. 1e shows a significant increase in the Mg concentration due to the high Mg concentration in the leached ferrochrome slag. The lowest Mg concentration was observed at the highest pH of 6 this may be attributed to part of the Mg precipitating due to reduced solubility of Mg at elevated pH [19].

### 3.2 Experimental design

Table 4 shows the experimental design for the recovery of phosphorus for the three variables (pH; N:P; Mg:PO_4_) investigated.

**Table 4:** Experimental design.

Run	pH	N:P	Mg:PO_4_	^X^pH	^X^N:P	^X^Mg:PO_4_	Y_o_
		ratio	ratio				PO_4_recovery_ (%)
1	7	1	1	−1	−1	−1	28.7
2	10	1	1	1	−1	−1	71.5
3	7	3	2	−1	1	−1	50.2
4	10	3	1	1	1	−1	73.4
5	7	1	2	−1	−1	1	16.4
6	10	1	2	1	−1	1	82.07
7	7	3	1	−1	1	1	24.6
8	10	3	2	1	1	1	89.6
**Axial Points**							
9	5.98	2	1.5	−1.682	0	0	0.87
10	11.02	2	1.5	1.682	0	0	84.3
11	8.5	0.32	1.5	0	−1.682	0	39.6
12	8.5	3.68	1.5	0	1.682	0	89.07
13	8.5	2	0.66	0	0	−1.682	64.2
14	8.5	2	2.34	0	0	1.682	85.6
**Centre Points**							
15	8.5	2	1.5	0	0	0	86.04
16	8.5	2	1.5	0	0	0	86.28
17	8.5	2	1.5	0	0	0	87.83
18	8.5	2	1.5	0	0	0	86.01
19	8.5	2	1.5	0	0	0	87.87
20	8.5	2	1.5	0	0	0	86.62

The phosphorus recovery (α) was deduced using equation 1;

(equation 1) $$\alpha =\frac{m_{o}-m_{f}}{m_{o}}$$

The symbols, $m_{o}$ and $m_{f}$ represent the initial and final concentrations of phosphorus in (g/L).

**Fig. 2:**
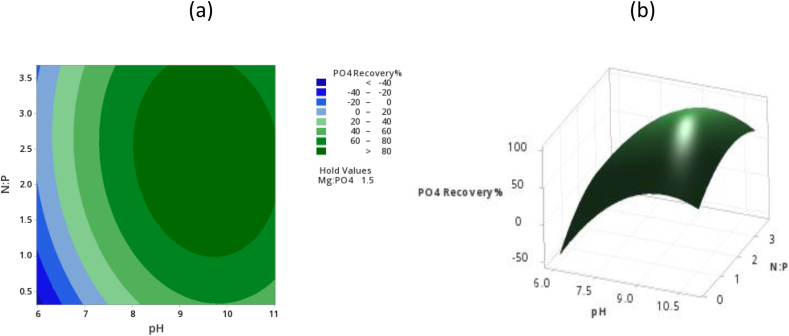
(a): Response surface plot phosphorus recovery% against pH and N:P (b): Contour plot phosphate reduction% against pH and N:P.

The interactive effects of varying pH and N:P simultaneously are shown in Fig. 2a and (b), both parameters have positive effects on phosphorus recovery. An increase in N:P molar ratio increases the buffer capacity of solutions as well as the kinetics of struvite formation which favours the recovery of phosphate. In addition, the enhanced buffer capacity of the solution lowers the required pH for nucleation of struvite crystals which accelerates the precipitation process. Fig. 2 (a) also shows that at a pH below 7 there was minimal phosphate recovery less than 20%, whereas above a pH of 11, the phosphate recovery was decreased from above 80% recovery. The pH is an important governing factor for struvite precipitation as it affects the species and the quantity of phosphates and ammonium in the solution. A sharp increase in the recovery of phosphorus was observed from a pH of 6 to 11 with a substantial amount of phosphorus recovered at a pH between 8 and 11, at this range struvite is least soluble. The highest phosphorus recovery was 89.6% although it should be noted that P exists in solution in many forms, and they are in dynamic equilibrium. The ionic species considered are PO_4_^3−^, HPO_4_^−^ H_2_PO_4_^−^ and the dissolved species is H_3_PO_4_. Consequently, other crystals can form such as newberyite Mg(PO_3_OH).H_2_O bobieritte Mg_3_(PO_4_)_2_.8H_2_O cattiite Mg_3_(PO_4_)_2_.22H_2_O [20].

**Fig. 3:**
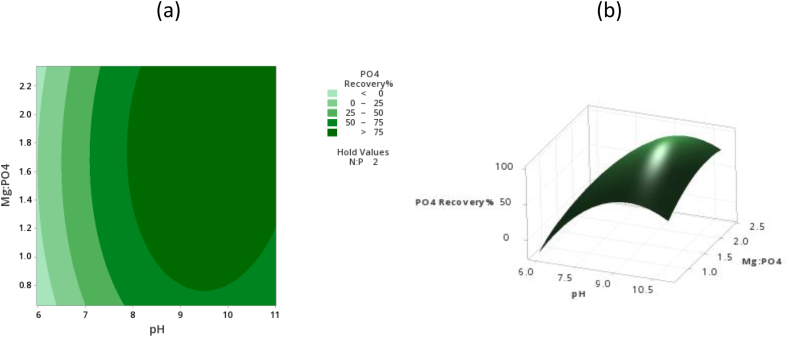
(a): Response surface plot phosphorus recovery% against pH and Mg:PO_4_ (b): Contour plot phosphate reduction% against pH and Mg:PO_4_.

Fig. 3 (a) and (b) shows the interaction effects between Mg:PO_4_ and pH increasing the Mg:PO_4_ ratio increases the ionic strength of the solution and the net charge repulsion is decreased resulting in the potential attraction of struvite forming ions which decrease the solubility and inturn favours the recovery of phosphate. Since the source of Mg contains foreign ions overdosing the Mg can result in a high concentration of the counterions which interfere with struvite precipitation resulting in a slight antagonistic effect on the phosphorus recovery. Fig. 3a also shows that the contour line is quite dense indicating that pH has a greater degree of influence on the P recovery. Above a pH of 8 and Mg:PO_4_ ratio of 1, the phosphorus recovery was high and remained relatively stable without any reduction. This was primarily due to the combined effect of reduced solubility of phosphorus above a pH of 8 and improved kinetics of the struvite precipitation process at high Mg:PO_4_ ratios. Besides this, the increased Mg:PO_4_ has a positive impact on the ionic strength of the solution, such that increasing the ionic strength can lead to a reduction in the double layer thickness around the crystal and enhance the mass transfer of monomers to the crystal surface favouring the recovery of phosphorus.

**Fig. 4:**
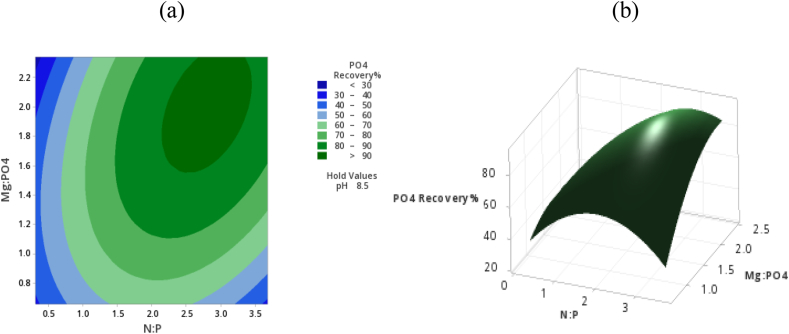
(a): Response surface plot phosphorus recovery% against N:P and Mg:PO_4_ (b): Contour plot phosphate reduction% against N:P and Mg:PO_4_.

Fig. 4a and (b) shows the interaction effect of N: P and Mg: PO_4_ the contour plot shows a low phosphate recovery at a N:P molar ratio of less than 1 regardless of an increase of Mg:PO_4_ ratio, this shows that limiting other desired constituents at levels that do not satisfy the minimal molar ratio of 1:1:1 for Mg:N:P is detrimental towards phosphate recovery. This agrees with findings by Ref. [21] that treatment of wastewater with a low phosphate concentration is not economically rewarding. In addition, the plots show that at a constant pH of 8.5 and an N:P molar ratio above 1.5 and Mg:PO_4_ molar ratios above 1, a significant phosphate recovery was observed. On the other hand, increasing the N:P ratio above about 3.2 had an inconsiderable effect on the recovery of phosphorus. More importantly, increasing the N:P ratio at constant pH reduced the recovery of phosphorus. This may be attributed to that the kinetics of struvite crystallization will not be significantly improved when particularly pH is restricted to a constant value [22]. There was a notable reduction in the phosphorus recovery above a Mg:PO_4_ of 2.2 and N:P ratio of 3.0 which may be attributed to the reduction in the activity of precursor ions of the struvite process with high ionic strength due to forming complexes with counterions in the ferrochrome slag [3].

The experimental results in Table 2 were used to deduce Equation (2). Equation (2) shows the fitted second-order model obtained through response surface modeling is shown in Eq. (1);

$$y=-668.6+141.6x_{1}+42.5x_{2}+32x_{3}-7.362x_{1}^{2}-8.88x_{2}^{2}+$$

(Equation 2) $$-20.56x_{3}^{2}-1.69x_{1}x_{2}+2.24x_{1}x_{3}+10.88x_{2}x_{3}$$

where: x_1_ – is the coded value for pH

x_2_ – is the coded value for N:P
x_3_ – is the coded value for Mg:PO_4_
y – is the response (PO_4_recovery_%)

**Fig. 5:**
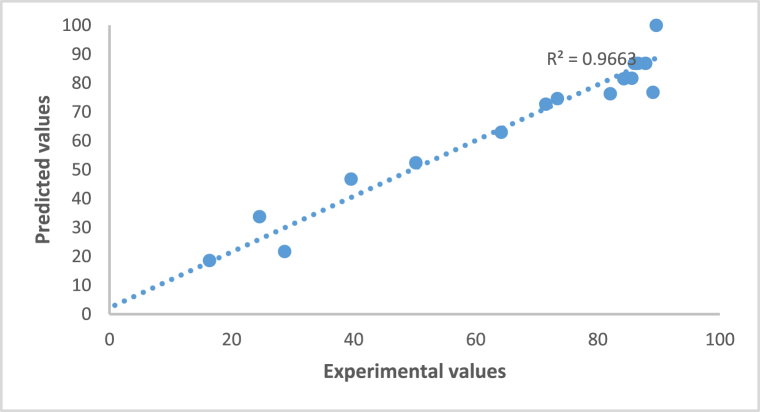
Normal probability plot.

The normal probability plot shown in Fig. 5 of residuals is another useful tool to check the model adequacy ensuring that the experimental data was adequately adapted to the model, detecting and ensuring systematic departures from the assumptions that the errors are normally distributed and the variance is homogeneous. In this scenario data are plotted near a straight line. Residuals are approximately linear confirming that they are normal and independently distributed.

The determination coefficient R^2^ was 0.97 which indicated that more than 95% of the variations could be explained by the model and there was a good correlation between the experimental and predicted values.

## 4 Analysis of variance

The deduced data was evaluated using analysis of variance (ANOVA) to fit a quadratic model and to assess the quality of the fit. At the 95% confidence level, the significance of the positives coefficient parameters was determined using theP values which are all less than 0.05 as shown in Table 5 indicating high dependence of phosphorus recovery on the investigated parameters [16]. Whilst the F model value was 140.09 shown in Table 6 indicating the high significance of the investigated parameters and reliability of the regression model for predicting phosphorus recovery.

**Table 5:** Tests on the individual variables quadratic model.

	Coefficients	Standard Error	t Stat	*P*-value
Intercept	86.92	2.95	29.47	0
pH	24.67	1.96	12.61	0
N:P	8.96	1.96	4.58	0.001
Mg:PO_4_	5.57	1.96	2.85	0.017

**Table 6:** Analysis of variance (ANOVA) for the fitted model.

	df	SS	MS	F
Error	10	528.3	52.3	
Lack of Fit	5	519.3	103.85	140.09
Pure Error	5	3.7	0.74	

## 5 Conclusion

The study demonstrated the ability to use the ferrochrome slag as a source of iron ions required for coagulation which is a pre-treatment process before struvite precipitation and simultaneously being an alternative magnesium source for struvite precipitation. The high recovery of phosphorus of 89.6% indicates that ferrochrome slag can be an adequate magnesium source for struvite precipitation. The investigated parameters (pH; N:P; Mg:PO_4_) showed that they all have a significant impact on the phosphorus recovery deduced from the ANOVA which had all p values < 0.05. Although the process was conducted under controlled conditions further studies must be conducted at a large scale to verify the impact of scaling up from which a detailed costing can be determined.

## Author contribution statement

L B Moyo: Conceived and designed the experiments; Performed the experiments; Analyzed and interpreted the data; Wrote the paper.

G.S. Simate, T. A. Mamvura, G. Danha: Analyzed and interpreted the data; Contributed reagents, materials, analysis tools or data; Wrote the paper.

## Data availability statement

Data will be made available on request.

## Declaration of interests

The authors declare that they have no known competing financial interests or personal relationships that could have appeared to influence the work reported in this paper.
